# Therapeutic Potential for Sphingolipids in Inflammatory Bowel Disease and Colorectal Cancer

**DOI:** 10.3390/cancers16040789

**Published:** 2024-02-15

**Authors:** Keila S. Espinoza, Ashley J. Snider

**Affiliations:** 1 Department of Physiology, University of Arizona, Tucson, AZ 85721, USA; espinoza5@arizona.edu; 2 School of Nutritional Sciences and Wellness, University of Arizona, Tucson, AZ 85721, USA; 3 University of Arizona Cancer Center, University of Arizona, Tucson, AZ 85721, USA

**Keywords:** inflammatory bowel disease, colitis-associated cancer, colorectal cancer, sphingolipids, ceramide, sphingosine-1-phosphate, inflammation

## Abstract

**Simple Summary:**

Patients with inflammatory bowel disease (IBD) suffer a lifelong disease of gastrointestinal inflammation. Furthermore, those with IBD demonstrate high risk in developing colorectal cancer (CRC). Rising cases of pediatric and adult onset IBD highlight a growing concern for addressing and alleviating inflammation to not only improve quality of life, but also curb the development of CRC. Disrupted sphingolipid metabolism has been implicated in the promotion of inflammation in IBD, creating an environment favoring the development of CRC. This has led to the examination of enzymes and receptors in sphingolipid metabolism as therapeutic targets for IBD and CRC. This review discusses modulators of key sphingolipid targets and their potential in attenuating gastrointestinal inflammation from cell culture models to patient trials.

**Abstract:**

Inflammatory bowel disease (IBD), characterized by chronic inflammation in the intestinal tract, increases the risk for the development of colorectal cancer (CRC). Sphingolipids, which have been implicated in IBD and CRC, are a class of bioactive lipids that regulate cell signaling, differentiation, apoptosis, inflammation, and survival. The balance between ceramide (Cer), the central sphingolipid involved in apoptosis and differentiation, and sphingosine-1-phosphate (S1P), a potent signaling molecule involved in proliferation and inflammation, is vital for the maintenance of normal cellular function. Altered sphingolipid metabolism has been implicated in IBD and CRC, with many studies highlighting the importance of S1P in inflammatory signaling and pro-survival pathways. A myriad of sphingolipid analogues, inhibitors, and modulators have been developed to target the sphingolipid metabolic pathway. In this review, the efficacy and therapeutic potential for modulation of sphingolipid metabolism in IBD and CRC will be discussed.

## 1. Introduction

### 1.1. IBD and Characteristics between CAC and CRC

Inflammatory bowel disease (IBD) encompasses Crohn’s disease (CD) and ulcerative colitis (UC), with CD occurring throughout the entire gastrointestinal tract, while UC is restricted to the colon. The causes of IBD are multifactorial and dependent on a wide variety of influences such as genetics, diet, alterations to the gut microbiome, and immune cell infiltration [1]. In 2015, it was estimated that up to 1.3% of the U.S. adult population, equating to over 3 million individuals, were diagnosed with IBD [2]. Studies estimate that cases of IBD have doubled in both pediatric and adult populations from 2007 to 2016 in the United States, and that the number of diagnoses will continue to rise [3]. Individuals with IBD, particularly UC, have an increased risk of developing colitis-associated colon cancer (CAC), where the lifetime risk of developing CAC is four to ten times greater compared to the sporadic development of colorectal cancer (CRC). In addition, CAC often develops at a younger age [4,5]. There are notable differences in the development and progression, involvement of inflammation, and severity between CRC and CAC. Mutations in the tumor suppressor gene adenomatous polyposis coli (APC) occur most commonly in CRC rather than CAC [6]. Furthermore, patients with CAC have a mortality rate 1.7 times greater than those with CRC [7]. The aforementioned rise of pediatric IBD is of great concern, as it results in patients facing severe issues, complications, or CAC in individuals as young as 30 to 45 years old. Thus, novel therapeutic targets for patients with IBD prior to the development of CAC are crucial.

### 1.2. Sphingolipid Metabolism

**Figure 1:**
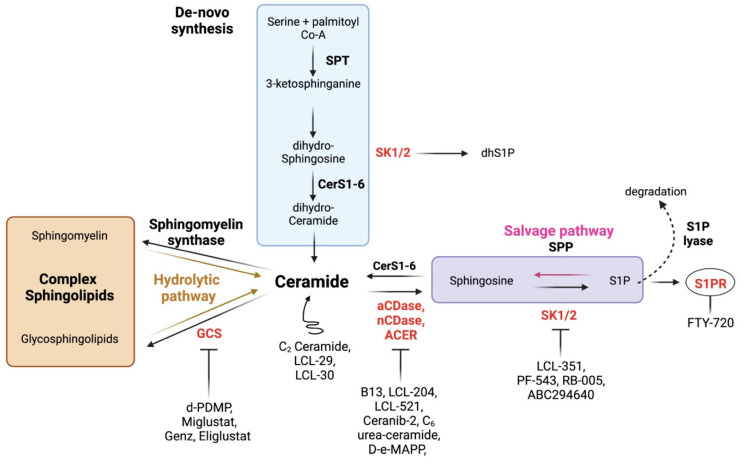
Sphingolipid metabolism and potential interventions. Ceramide is at the center of sphingolipid metabolism, and can be generated via the de novo, hydrolytic, or salvage pathways. Ceramide plays several roles in arresting cellular activity, and can be utilized to generate complex membrane sphingolipids, or deacylated to generate sphingosine and eventually the potent signaling molecule S1P. With the exception of S1PR, various enzymes (in red), which have been implicated in inflammation, can be targeted with inhibitors. S1PRs, the receptors for S1P, can be inhibited with specific inhibitors and/or downregulated by S1PR modulators. Lastly, ceramide analogues increase ceramide pools to drive its biological effects.

Sphingolipids are a family of bioactive lipids with roles in the plasma membrane, endoplasmic reticulum (ER), and both intra- and extracellular signaling. Sphingolipids are generated de novo in the ER by the condensation of serine and palmitoyl co-A by serine palmitoyl transferase (SPT), generating 3-keto-dihydrosphinsosine, which is rapidly reduced to dihydrosphingosine (dhSph), which may then also be phosphorylated by sphingosine kinases (SKs) to generate dihydrosphingosine-1-phophate (dhS1P). One of six ceramide synthases (CerS), each with *N-acyl* chain length preference, can also acylate dhSph-generating dihydroceramides (dhCer). DhCer is then desaturated by dihydroceramide desaturases (DES1 and DES2) into the central sphingolipid Cer. Multiple enzymes can then act on Cer to generate more complex sphingolipids, including sphingomyelin (SM) and glucosylceramide (GluCer). Ceramidases (CDases) de-acylate ceramide to produce sphingosine, which can then be phosphorylated by SKs to generate sphingosine-1-phosphate (S1P). S1P is a potent signaling molecule that binds to one of five G-protein-coupled S1P receptors (S1PRs) and can be dephosphorylated to sphingosine by sphingosine phosphate phosphatases (SPPs) or irreversibly degraded by S1P lyase (SPL). Cer can also be generated by both the hydrolytic or salvage pathways by the action of sphingomyelinases (SMases) or via re-acylation of sphingosine by CerS, respectively (Figure 1).

### 1.3. Altered Sphingolipid Metabolism in IBD, CAC, and CRC

**Figure 2:**
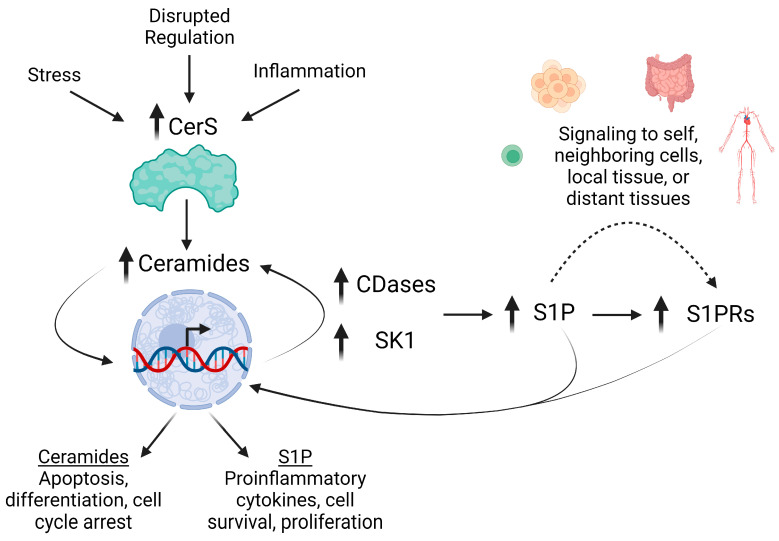
Altered sphingolipid metabolism in IBD and CRC promote inflammation. Early events in IBD and cancer trigger the over-expression of CerS, increasing the generation of ceramide species. Elevations in Cer can then drive apoptosis, differentiation, and cell cycle arrest. However, Cer also influences expression of CDases and SK1. Distinct pools of Cer can then be deacylated to generate sphingosine and phosphorylated into S1P. S1P signaling is mediated via autocrine to paracrine signaling via S1PRs, which are also highly expressed during inflammation, increasing S1P signaling to promote proinflammatory cytokine secretion, cell survival, and proliferation.

Sphingolipids are involved in diverse biologic roles, including providing cellular structural integrity and acting as signaling molecules regulating apoptosis, proliferation, senescence, and inflammation [8]. Alterations to sphingolipid metabolism have been well implicated in IBD, CAC, and CRC. A study examining multiple cohorts of CRC patients found significant upregulation of various genes related to sphingolipid metabolism, the most prevalent being acid ceramidase (*ASAH1*), sphingosine kinases 1 and 2 (*SPHK1, SPHK2*), and S1PR1 and 4 (*S1PR1*, *S1PR4*) [9]. Furthermore, colon tissues from CRC patients exhibited elevated mRNA expression of CerS2, CerS5, and 6 [9,10]. The roles for sphingolipid-generating enzymes in IBD and CRC have been further investigated in cell culture and mouse models (Figure 2).

The generation of ceramide by CerS has been evaluated in numerous mouse models for IBD and cancer. Mice lacking CerS2 exhibited poor survival and were more susceptible to dextran sulfate sodium (DSS)-induced colitis [11]. Global loss of CerS4 similarly exacerbated azoxymethane/dextran sulfate sodium (AOM/DSS)-induced CAC in mice; however, loss of CerS4 in T cells alone reduced neutrophils in circulation and infiltration into colon tissue [12]. Despite the implications of CerS5 in CRC, global knockout of CerS5 exacerbated pathology scores and inflammation in mice with DSS-induced colitis, and increased tumor burden in AOM/DSS mice [13]. While C16 Cer levels were reduced in colon tissues of CerS5^−/−^ mice, S1P remained significantly elevated in the blood, which could have contributed to significant immune cell mobilization to the colon. Micro RNA (miRNA) 148a downregulated CerS5 and was reduced in patients with CRC, resulting in lack of CerS5 inhibition [14]. Mice deficient in miR-148a demonstrated dysbiosis and increased tumorigenesis in the AOM/DSS model of CAC. This was reversed upon treatment with short hairpin RNA (shRNA) against CerS5. The expression of CerS5 in human CRC cell lines is significantly reduced after treatment with 5-flourocil (5-FU) and sensitizes the cells to apoptosis [15]. In contrast, Brachtendorf et al. demonstrated increased expression of CerS5 after 5-FU treatment in a p53-dependent manner, promoting autophagy and resistance to therapy [16], suggesting that CerS5 and Cer may play multiple roles dependent on the stage of cancer. Interestingly, knockdown of CerS5 in human T cell lymphoma cell lines ablated NFkB dependent activation [15], suggesting that sphingolipid metabolism in immune cells may provide additional therapeutic targets.

Though CerS5 and 6 have similar *N*-acyl chain-length preference in the generation of ceramide, some distinctions in their role in disease have been noted. Though studies have demonstrated increased CerS6 mRNA expression in patients with CRC, overexpression of CerS6 (or CerS2) led to decreased viability in several CRC cell lines [9]. Similar to global knockout of CerS5, total body loss of CerS6 exacerbated colitis pathology scores and increased neutrophil infiltration in mice with DSS-induced colitis [17]. In addition, intestinal epithelial cells (IECs) isolated from fat-mass-and-obesity-associated protein (FTO) conditional knockout mice were more susceptible to DSS-induced colitis and demonstrated decreased CerS6 expression [18]. Conversely, loss of CerS6 in splenocytes in a T cell transfer model of colitis, protected from disease [19]. Reduced CerS6 expression has been shown to decrease apoptosis in response to TNF-related apoptosis-inducing ligand (TRAIL) in CRC cell lines [20]. Despite cleavage of caspase 3, which would typically result in apoptosis, the downregulation of CerS6 expression prevented caspase 3 translocation into the nucleus. These studies suggest that disruptions in CerS expression may play critical roles in inflammatory responses in colitis and CAC, but that the exact implications are still to be elucidated.

Degradation of ceramide by ceramidases (CDase) has also been implicated in IBD, CAC, and CRC. Patients with IBD exhibited higher levels of acid ceramidase (aCDase) in colon biopsies compared to normal controls [21]. Mice lacking aCDase in myeloid cells, but not in intestinal villi, were protected from DSS-induced colitis, further suggesting that sphingolipid metabolism in immune cells may prove to be a vital therapeutic target. Loss of neutral ceramidase (nCDase) [22,23] or alkaline ceramidase 3 (ACER3) increased susceptibility to colitis, and ACER 3 significantly increased CAC in an AOM/DSS model [24]. Interestingly, loss of nCDase protected mice from development of CRC upon AOM administration [23], suggesting distinct impacts for sphingolipid enzymes in colitis-associated and sporadic CRC. Links between ceramide generation and degradation have also been shown in CRC cell lines. Overexpression of CerS6 in CRC cells lines significantly increased the expression of aCDase in a JNK-dependent manner [25], suggesting the expression of sphingolipid enzymes may be co-regulated. Together these studies suggest that ceramidases may play cell-specific roles in the regulation of inflammation and development of CAC/CRC.

Cer can also be utilized for complex sphingolipids such as SMs, which make up various membranous components of cells. Mice lacking SM synthase 2 (SMS2) were protected from immune cell infiltration after DSS-induced colitis and AOM/DSS-induced tumorigenesis [26]. Furthermore, several aberrant signaling pathways which promote inflammation and cancer, such as phospho-ERK1/2 and STAT3, were significantly reduced in colonic tissue. Sphingomyelinases (SMases) cleave SMs to regenerate Cers. Alkaline SMase (alkSMase) activity is significantly reduced in patients with UC, and is lowest in areas of dysplasia [27]. Mice lacking alkSMase were more susceptible to DSS-induced colitis associated inflammation [28]. Similarly, mice lacking acid SMase (aSMase) demonstrated increased inflammation after infection of *Citrobacter rodentium* in a model of infectious colitis. However, pharmacological inhibition of aSMase by SMA-7 [29] or desipramine [30] in mouse models of DSS-induced colitis significantly reduced inflammatory cytokines and tissue damage. These studies implicate SMSs and SMases as targets for IBD, but suggest that inhibition of sphingolipid enzymes, rather than its total loss, may best demonstrate a therapeutic effect.

SKs and S1P have been implicated in colitis, CAC, and spontaneous CRC. SK1 is highly expressed in patient samples with colitis [31] and CRC, and has been shown to be more highly expressed in CAC tissues than in CRC [32,33]. Early work examining SK1 expression in AOM-induced CRC in rats demonstrated increased SK1 expression in CRC tumors [33]. While mice lacking SK1 developed fewer tumors and exhibited a significant reduction in multiple inflammatory markers after AOM/DSS-induced CAC, as well as impaired immune cell infiltration in DSS induced colitis [31]. Loss of SK1 specifically in the intestinal epithelium has also been shown to protect against CAC in the APC^Min/+^ mice treated with DSS [34]. Interestingly, mice lacking SK2 developed more tumors in the AOM/DSS model of CAC, and more severe colitis [35]. The lack of SK2 resulted in elevated SK1expression, and increased S1P in the blood and tissue. Loss of S1P-degrading enzymes has also been implicated in IBD and CRC. Specific deletion of SPL in intestinal tissues resulted in increased inflammation and tumorigenesis in the AOM/DSS model of CAC [36]. In addition, SPP2 is highly expressed in patients with UC and in mice following DSS-induced colitis [37]. Interestingly, mice lacking SPP2 exhibited less DSS-induced weight loss, reduced colitis scores, and decreased proinflammatory cytokine secretion; however, mice lacking SPP1 demonstrated enhanced colitis severity. These studies suggest that the enzymes that generate and/or degrade S1P may play significant roles in IBD and CAC.

S1PRs are involved in immune cell trafficking, activation, and cytokine secretion and have been extensively investigated in intestinal inflammation and cancer. S1PR1 is highly expressed in the colonic vasculature of patients with UC [38], while S1PR2 has been shown to be down regulated in CRC patients [39]. Moreover, modulation of S1PR1 expression has been down to protect from CAC in mice [35]. However, mice lacking S1PR1 demonstrated impaired intestinal permeability and increased bleeding after DSS-induced colitis [38]. Loss of S1PR2 mice resulted in higher tumor incidence in both the APC^+/min^ and AOM/DSS models for CRC and CAC [39]. Conversely, mice deficient in S1PR4 were protected from DSS-induced colitis, ablating proinflammatory cytokines and reducing IL-17+ T cells [40]. Loss of S1PR4 was further assessed in AOM/DSS-induced CAC, and mice demonstrated significant reductions in tumor size and significantly increased CD8+ T cells [41]. Significant progress in the modulation of S1PR signaling has led to FDA approval of S1PR modulators for UC (discussed in *S1PR Modulators FTY-720: Clinical Trials*).

The disruption in sphingolipid metabolism measured in IBD and CRC has given rise to numerous studies investigating inhibitors/modulators of these pathways in inflammation and tumorigenesis. This review will focus on the therapeutic potential for sphingolipids and their metabolizing enzymes as targets in alleviating IBD, preventing CRC, and sensitizing cancer cells to apoptosis (references summarized in Table 1). As this Special Issue of Cancers is in honor of Drs. Lina Obeid and Mark Kester, we have placed emphasis on acid ceramidase and sphingosine kinases to highlight their work.

## 2. Inhibitors of Sphingolipid Metabolism

### 2.1. Glycosphingolipid (GSL) Inhibitors

The accumulation of GSLs are pathological in various contexts, and are typically implicated in lysosomal storage disorders and neurodegeneration.

The application of glucosylceramide synthase (GCS) inhibitors has been assessed in CRC, but not colitis. CRC patients who exhibit high expression of *UGCG*, the gene encoding GCS, have increased risk of mortality [42]. Jennemann et al. demonstrated treatment of human CRC cell lines with either Genz or Miglustat resulted in cell cycle arrest and inhibited the development of spheroids, phenotypic of de-differentiated cells. Interestingly, inhibition of GCS did not increase Cer levels, but instead increased SM. This also examined inhibition of GCS in the AOM/DSS model of CAC, where treatment with Genz in the diet (0.225% of diet) reduced tumor number and size. Short-chain enoul-CoA hydratase (ECHS1), involved in fatty acid metabolism, has been found to upregulate *UGCG*, resulting in the increased synthesis of GCS and complex GSL [43]. Ceramide levels were increased in CRC cells overexpressing ECHS1 and treated with Eliglustat. This led to activation of autophagic and apoptotic pathways. However, in contrast to the study by Jennemann et al., Eliglustat did not alter SM content. In vivo, dual treatment with oxaliplatin and Eliglustat (75 mg/kg) reduced tumors in mice bearing CRC xenografts overexpressing ECHS1. These data suggest that the inhibition of GCS may serve as promising therapeutic target for CRC.

### 2.2. CDase Inhibitors

#### 2.2.1. Acid Ceramidase (aCDase)

Expression of aCDase has been shown to be increased in patients with IBD and CRC [21]. Though not extensively studied in colitis, CAC, or CRC, aCDase inhibitors have been significantly implicated in prostate, breast, and HNSCC cancers. These studies are described briefly here.

##### B13

B13, a novel aCDase specific inhibitor, was first tested on human keratinocytes and melanomas. B13-induced apoptosis in a dose- and time-dependent manner due to Cer accumulation [44]. However, cells overexpressing BCL2 escaped B13-induced apoptosis. Although B13 demonstrated promise, many analogues of B13 quickly rose and outpaced the parent compound in the following years.

##### LCL-204

A subsequent analogue of B13, LCL-204 has provided new insight into the inhibition of aCDase in cancer, and has been studied most extensively in models of prostate cancer. LCL204 treatment in prostate cancer cell lines significantly degraded aCDase, destabilized lysosomes, and altered mitochondrial membrane potential [96]. The use of this aCDase inhibitor has also been assessed for efficacy in combination with various gene therapies. When used in combination with either gene therapy Apoptin or FasL treatment, LCL-204 reduced tumor burden and increased survival (without inducing overt toxicity) in athymic^nu/nu^ mice bearing prostate cancer (75 mg/kg) [45] and HNSCC xenografts (50 mg/kg) [46].

##### LCL-521

As a more recently developed B13 analogue, LCL-521 was first synthesized in 2014 and characterized in human breast adenocarcinoma cell lines [47]. LCL-521, a pro-drug that is only active in the lysosome, demonstrated more potent inhibition of aCDase when compared other B13 analogues. LCL-521 significantly decreased Sph content and increased degradation of aCDase, but did not lead to sustained destabilization of lysosomes. Further analysis revealed that LCL-521 sensitized Tamoxifen resistant human breast adenocarcinoma cells to treatment, and that at higher doses would inhibit DES1, the enzyme responsible for the desaturation of dhCer [48,49]. LCL-521 has since been investigated in combination with various chemotherapies. aCDase is highly expressed in radiation-resistant prostate cancer cell lines. Moreover, athymic^nu/nu^ mice bearing prostate cancer xenografts demonstrated significantly longer survival when treated with LCL-521 (75 mg/kg) and ionizing radiation when compared to either treatment alone [97].

##### Ceranib-2 (C-2)

Ceranib was first synthesized and described by Draper et al. in 2011 [50]. As a novel analog to Ceranib-1, Ceranib-2 (C-2) demonstrated a greater affinity for inhibiting aCDase activity in SKOV3 human ovarian adenocarcinoma cell lines. C-2 treatment increased de novo synthesis of dhC16 Cer, with a notable lack in dhS1P generation, when compared to Ceranib-1. Treatment with C-2 significantly reduced tumor volumes in Balb/c mice bearing mammary adenocarcinomas, indicating C-2 has potential anti-cancer therapy. Further studies in cell culture have demonstrated efficacy of C-2 in human glioma [51], prostate [52,53], breast [54,55], CRC [56], lung [57], and cervical cancer [58] cell lines. However, the therapeutic potential for C-2 alone may be hindered due to solubility issues. The engineering of nanotechnology delivery systems for sphingolipid modulators, pioneered by Dr. Mark Kester [98,99], has allowed for improved delivery of often insoluble or poorly soluble molecules. Nanocarrier delivery of C-2 in colorectal adenocarcinomas, cervical adenomas, and prostate cancer cell lines has demonstrated the ability to induce apoptosis [53,58]; however, not to the same extent as C-2 alone [58]. Animal studies have yet to be described with C-2, leaving a notable gap in the knowledge about the in vivo efficacy of nanocarrier delivery or potential side effects of C-2.

The above-mentioned studies highlight the significance for aCDase in cancer, though there are limited studies for this enzyme in colitis, CAC, and CRC. The significance of aCDase in genetic models of colitis and CAC, as well as tissues from human patients [21], suggests significant therapeutic potential for aCDase in colitis and CAC.

#### 2.2.2. Neutral Ceramidase (nCDase)

##### C_6_ urea-Cer

Genetic deletion of nCDase resulted in increased susceptibility to chemical-induced colitis [22], but prevented tumor development in the AOM model for CRC [23]. These studies led to the investigation of C_6_ urea-Cer, a nCDase inhibitor, in both cell culture and animal models of CRC. C_6_ urea-Cer treatment in human CRC cell lines (HT-29 and HCT116) elevated caspase 3, LCL3-I, and LCL3-II, leading to apoptosis and cell cycle arrest [23]. Further, treatment of these CRC cell lines with C_6_ urea-Cer reduced β-catenin and phospho-ERK, both markers for cell survival and proliferation. Tumor volume and markers were significantly decreased in mice treated with 5 mg/kg C_6_ urea-Cer, while tumor Cer levels were increased. Treatment with C_6_ urea-Cer reduced circulating neutrophils, but did not alter body weight, suggesting potential effects on circulating immune cells, but not overt toxicity. In cell culture models, C_6_ urea-Cer treatment prevented the phosphorylation of AKT and GSK3β, resulting in decreased β-catenin expression and cell cycle arrest [59]. These studies highlight the significance of nCDase in CRC, and have spurred interest in finding novel nCDase inhibitors [100]. While these novel analogues have been identified, their potency and efficacy in vivo have yet to be published.

#### 2.2.3. Alkaline Ceramidase (ACER)

##### D-e-MAPP

Though not yet used in IBD or CRC, D-e-MAPP was first synthesized in 1996. Two enantiomers, D and L, were first examined as CDase inhibitors, where D-e-MAPP demonstrated significantly reduced cell growth and increased Cer levels in human leukemia cells [60]. It was further determined that D-e-MAPP inhibited aCDase and ACER, with greater affinity for the latter. The therapeutic implications of D-e-MAPP in IBD and CRC have yet to be determined.

### 2.3. Sphingosine Kinase (SK) Inhibitors

Significant work from the laboratory of Dr. Lina Obeid implicated sphingolipids in colitis, CAC, and CRC, with the initial studies aimed at the roles for SK1 [31,33,101]. These studies and others have demonstrated increased SK1 expression and generation of S1P in humans and in animal models, therefore suggesting significant therapeutic potential for inhibitors of SK.

#### 2.3.1. SK1

##### LCL-351

The novel SK1 inhibitor LCL-351 was first synthesized and characterized in colitis in 2017 [61]. This inhibitor exhibited 10-fold selectivity for SK1 over SK2. This selectivity was further demonstrated by significant decreases in S1P in mouse embryonic fibroblasts (MEFs) from wildtype and SK2 deficient mice treated with LCL351, but not MEFs from SK1 deficient mice. In vivo, LCL351 (6 mg/mg) was rapidly cleared from circulation, but remained in tissues up to 24 h without inducing toxicity. In an animal model of acute colitis (5% DSS), LCL351 significantly reduced S1P in the circulation, prevented loss of red blood cells and reduced splenomegaly. Furthermore, treatment with LCL351 significantly reduced neutrophil infiltration in the colon. These studies augmented the significance of previous findings where total body genetic deletion of SK1, as well as loss of SK1 in bone-marrow-derived cells, resulted in decreased neutrophil recruitment to the colon [31,102].

##### PF-543

PF-543 was developed in 2012 as a novel SK1-specific inhibitor, and tested for efficacy across various cancerous cell lines [62]. PF-543 preferentially inhibits SK1, reducing S1P and elevating Sph with an IC_50_ in vitro and in cells of ~1–2 nM. Human CRC cell lines treated with PF-543 (1–10 µM) exhibited significant loss of cell viability, inhibition of colony formation, and cell death; however, this varied depending on CRC cell line [63]. Though the IC_50_ for PF-543 ~1–2 nM, and initial studies demonstrated no off-target inhibition of protein or lipid kinases (85 tested at a dose of 10 µM) [62], the possibility exists that the higher doses utilized in the literature may alter cellular viability due in part to off-target effects. In an animal model of acute colitis, PF-543 (10–30 mg/kg) attenuated loss of body weight, maintained colon length, and reduced levels of proinflammatory cytokines in the colon [64]. Treatment with PF-543 (25 mg/kg) also significantly reduced tumor volume and increased survival in mice bearing CRC xenografts [63]. These data suggest promising findings in both in vitro and in vivo models for PF-543 in IBD and CRC.

##### RB-005

Through the success of PF-543, RB-005 was developed as a novel derivative inhibitor of SK1. Human CRC cell lines treated with RB-005 exhibited reduced proliferation and increased apoptosis [65]. The inhibition of SK1 resulted in reduced S1P content while increasing Cer. Further analysis found that treatment increased the activity of tumor suppressor PP2A, resulting in decreased phosphorylation of AKT and ERK pathways. Interactions between Cer and protein phosphatases and decreased cellular signaling are well known [59,103,104]. Therefore, it is possible that the increased Cer associated with RB-005 may have additional indirect effects on cell signaling pathways, potentially via activation of protein phosphatases. Though still novel and requiring additional studies, these data indicate promising use as a therapeutic against CRC.

Collectively, these studies suggest that inhibition of SK1 protects from acute colitis, and may serve as a potential therapeutic target in both CAC and CRC.

#### 2.3.2. SK2 Inhibitors

##### ABC294640

ABC294640 acts as a competitive inhibitor of SK with preferential inhibition of SK2. In addition, ABC294640 has been shown to result in degradation of DES1 and SK1 [66]. Human CRC cell lines dosed with ABC294640 in combination with Vemurafenib, a common chemotherapeutic used in melanoma, demonstrated significant inhibition of SK2, reduced S1P content, and induction of apoptosis [67]. Further analysis revealed significant reductions in the phosphorylation and/or protein content of AKT, ERK1/2, and MEK1/2, suggesting that treatment restricted aberrant cell signaling.

Use of ABC294640 (50 mg/kg) in the DSS-induced model of colitis (2% DSS) resulted in retention of healthy tissue, ablation of cytokine expression, reduced neutrophil infiltration, and reductions in colonic S1P levels [68]. ABC294640 also reduced inflammation and neutrophil infiltration in the TNBS model for IBD in both mice and rats [69]. In an AOM/DSS model of CAC, ABC294640 (50 mg/kg) elevated levels of Sph, but not S1P, in the colon, and reduced tumor incidence [70]. In this model, inhibition of SK induced the upregulation of tumor suppressor Beclin-1, as well as reduced phosphorylation of AKT and ERK1/2, mimicking previous cell culture data [70].

A phase I clinical trial for ABC294640 (Opaganib) assessed the treatment of 16 patients with various solid tumors, including 6 patients with CRC [71]. The patients assessed in this study were treated with 250 mg, 500 mg, and 750 mg, with the highest dose inducing nausea and vomiting in one patient, and two patients were unable to complete the first cycle due to drug-related toxicity. One patient (7%) exhibited a partial response, with 18 cycles of treatment prior to disease progression. Six patients (40%) demonstrated stable disease, with the longest progression-free survival being 336 days. The remaining 53% of patients were nonresponsive. The findings of this trial suggest that inhibition of SK may be a target in specific types of solid tumors including CRC, but further studies are necessary to determine the therapeutic potential.

## 3. Cer Analogues

Cer analogues have been utilized to mimic increased endogenous levels of Cer and inhibit Cer generating or degrading enzymes.

### 3.1. C_2_-Cer

C_2_-Cer was first synthesized in the late 20th century as one of the first Cer analogues. The short chain Cer readily passes through cell membranes, in comparison to LC and VLC Cer. Due to the focus of this review and the wide breadth of literature completed on C_2_-Cer, a selection of work dedicated to CRC is summarized.

The efficacy of C_2_-Cer against CRC was first demonstrated in 1998 in human CRC cell lines. C_2_-Cer demonstrated greater efficacy against CRC cell viability when compared to dihydro-C_2_-Cer [72]. This was later demonstrated to result from degradation of PARP and increased expression of pro-apoptotic genes, with a simultaneous downregulation of anti-apoptotic genes [73]. Interestingly, both sphingosine and dhSph, which are structurally similar to C_2_-Cer, elicited similar apoptotic effects [74].

Although C_2_-Cer functionally disrupts cancer cells resulting in apoptosis, in vivo work has not been completed in the context of CRC. However, the use of C_2_-Cer in illuminating cellular mechanisms and genetic targets in cancers renders it as useful investigatory tool in cell culture studies. The generation of novel Cer analogues, such as C_6_ and C_16_ analogues, have since superseded C_2_ and propelled Cer analogue use into in vivo models.

### 3.2. Catatonic Pyridinium C_6_ (LCL-29)

Though not studied in CRC, the modified catatonic-pyridinium-C_6_ Cer analogue, LCL-29, was first characterized by Senkal et al. in 2006 in head and neck squamous cell carcinomas (HNSCC) [75]. Treatment with LCL-29 (80 mg/kg) significantly reduced tumor size in athymic^nu/nu^ mice bearing HNSCC xenografts without inducing overt toxicity. LCL-29 accumulation was found to be tumor specific [105,106], and subsequent studies in HNSCC cell lines demonstrated cytochrome C release and ER stress, resulting in apoptosis and autophagy, respectively [76,77]. The success of LCL-29 drove the development of novel C_6_-Cer analogues, including the cationic C_6_-Cer, LCL-124. Studies in pancreatic cancer cell lines demonstrated similar mitochondrial-mediated apoptotic events as that of HNSCC [78]. Mice bearing pancreatic cancer xenografts treated with LCL-124 at 40 mg/kg exhibited a significant reduction in tumor size and an increase in survival.

### 3.3. Cationic C_16_ (LCL-30)

LCL-30 was first synthesized in 2006 as an additional cationic C_16_-Cer analogue to target mitochondrial metabolism. Similar to LCL-29 and LCL-124, LCL-30 accumulated in the mitochondria of human CRC cell lines, resulting in cytochrome C release [79]. LCL-30 (6 mg/kg) significantly reduced tumor volume and markers of proliferation in Balb/c mice bearing mouse orthotopic CT-26 tumors [80]. Plasma concentrations of LCL-30 peaked at 2 h, a demonstrating a somewhat shorter half-life compared to the C_6_-Cer analogue LCL-24 [75]. In addition, mice treated with LCL-30 also exhibited weight loss, suggesting the potential for toxicity. These studies suggest the potential for carbon length of the acyl-chain to alter sustained bioavailability.

## 4. S1PR Modulators

S1PRs have been implicated in the proliferation and migration of cancer cells, as well as modulation of immune cell trafficking. Several S1PR modulators have been investigated for their efficacy in IBD (recently reviewed in [107]), CAC, and CRC. SEW2871, an S1PR1 selective agonist, treatment in IL10-deficient mice resulted in improved barrier function and decreased epithelial cell apoptosis [108]. JTE-013, an S1PR2 antagonist, partially protected against colitis-like pathology in the DSS-induced colitis model [109], and it’s derivatives have been shown to enhance the effects of 5-FU in resistant CRC cell lines [110]. The S1PR agonist KRP-203 has been shown to protect from cytokines and recruitment of B and T cells to the colon in the IL10 deficient model of colitis [111]. Similarly, W-061, an additional S1PR agonist protected from DSS-induced colitis and reduced Th1 and Th17 recruitment to the colon [112]. These studies and others add to the large body of literature indicating the significance of S1PRs in intestinal inflammation and cancer.

### 4.1. FTY720

FTY720 was originally conceived for immunosuppressive use in organ transplantations and autoimmune diseases in 1996 through targeting of the S1PRs. Initial studies demonstrated improvement of skin grafts in rats by preventing tissue rejection [113]. Similar to the studies with JTE-013 derivatives, FTY720 treatment (4–20 µM) in HCT8/5FU CRC cells, which are resistant to chemotherapy 5-FU, decreased proliferation in a time- and dose-dependent manner [81]. Combination with the therapeutics doxorubicin and etoposide enhanced apoptosis and reduced the expression of resistance proteins. These data suggest FTY720 has potential therapeutic effects across several CRC cell lines.

The efficacy of FTY720 in cultured cells has also been translated to cultured patient samples. Treatment of cultured tumor samples from CRC patients resulted in reduced activity of tumor suppressor PP2A, as well as overexpression PP2A inhibitors SET and CIP2A [83]. In these samples, FTY720 significantly increased PP2A activity, reduced cell growth, and impaired colony formation. The effects of treatment were enhanced when combined with chemotherapies such as 5-FU, SN-38, and oxaliplatin. The success of FTY720 and the effects on PP2A have led to the development of additional derivatives, which have shown promise in human CRC cell lines [114]. However, long term use of FTY720 (10 μM) has been shown to promote autophagy and treatment resistance [82]. The responsiveness of patient samples to FTY720 suggests its use as a cancer therapeutic, and that combination with additional chemotherapies may provide patients with novel treatment plans.

### 4.2. In Vivo

Animal models have provided further insight into the therapeutic effects of FTY720 in colitis and cancer, and have elucidated the effects on immune cell modulation. Mice treated with FTY720 (1 mg/kg) prior to induction of acute colitis (3% DSS) demonstrated improvements to classical features of colitis, surprisingly due to elevations of S1P levels. These data may indicate an alternative role in S1PR modulation dependent on the timing of treatment and disease onset [84]. Balb/c mice in the acute model of colitis (3.5% DSS) treated with FTY720 (0.3 mg/kg) exhibited decreased neutrophil and CD4+ T cell infiltration to the colon [85]. FTY720 has also been used in combination with sulfasalazine in a TNBS model of IBD, resulting in the amelioration of colitis and inflammation [86]. This model achieved disease attenuation via maintaining populations of naïve T regs, which prevent helper 1 T (Th1)-cell-mediated colitis [87]. Additionally, CD4+ T cells isolated from treated animals secreted greater quantities of anti-inflammatory cytokines. T cell transfer models in Balb/c and SCID mice treatment with FTY720 (3 mg/kg) demonstrated significantly impaired colonic T cell infiltration [88]. Other studies on this model suggest that treatment (1 mg/kg) protected from colitis by sequestering T cells in secondary lymphoid organs [89]. Isolated T cells from treated animals demonstrated impaired secretion of proinflammatory cytokines, preventing the development of colitis associated Th1 and Th2 cells. Furthermore, transfer of activated T cells into treated mice continued to elicit immunosuppressive effects, demonstrating the ability to modulate preexisting activated immune cells. Treatment with FTY720 in the oxazolone mouse model of colitis (3 mg/kg) reduced *GATA-3* gene and T1/ST2 proteins, a Th2 cell transcription factor and protein involved in cytokine secretion, respectfully [90]. Similarly, rats treated (0.5 mg/kg) in the acetic acid model of UC demonstrated protection from colitis and inflammation [91]. These studies and others have led to clinical trials for S1PR modulators in CD and UC.

Potential pitfalls for the use of FTY720 in cancer were highlighted in mice bearing murine strains of CRC, lung cancer, or melanoma, in which treatment (1 mg/kg) promoted the growth of tumors by increasing immunosuppressive cells at the tumor microenvironment [92]. Assessments further determined that FTY720 may be functioning as a modulator for S1PR3. These studies highlight the importance of disease state, as well as time and dosing of FTY720, but reinforce the significance of S1PR modulation in inflammation.

### 4.3. Clinical Trials

Ozanimod (Zeposia^®^), an S1PR1 and 5 modulator, was FDA approved in 2010 for the treatment of multiple sclerosis [115]. It has since been in clinical trials testing for safety and efficacy in UC. The phase II TOUCHSTONE trial for long-term treatment of UC assessed 65 patients on placebo and 130 patients on low and high doses of Ozanimod [93]. After one year, up to 71% of patients demonstrated partial response, while 54% of patients achieved histologic and endoscopic remission. Following the initial Phase II, an open label extension study of four years was conducted. In this follow up study, Ozanimod sequestered lymphocytes and significantly reduced fecal calprotectin, a marker of intestinal inflammation. Less than 5% of patients demonstrated serious adverse events. The study concluded that Ozanimod provided favorable benefits to patients with moderate to severe UC. This group moved into an expanded phase III clinical trial, recruiting multiple cohorts consisting of over 300 patients with moderate to severe UC each over the span of one year [94]. Similar to the phase II trial, treatment significantly improved clinical response, endoscopic and mucosal healing, and achieved remission. Furthermore, treatment significantly improved maintenance of remission and increased achieved durable remission. Ozanimod was approved by the FDA for the treatment of UC in 2021.

Additional clinical trials for S1PR modulators are ongoing, including the Phase II clinical trials of Amiselimod, a novel S1P1R modulator. Eighty patients were recruited presenting with moderate to severe CD, and were split into placebo and treatment groups [95]. Treatment (0.4 mg) significantly reduced circulation of lymphocytes over time, consistent with previous S1PR modulators. However, treatment with Amiselimod reduced CD activity index score or induced remission in a small number of patients when compared to placebo. The most common side effects of treatment were headaches and progression of CD, reported by 10% and 15% of patients, respectively. At this time, there is no evidence demonstrating clinical efficacy of this modulator in CD. However, future studies with expanded patients and added measurements to assess efficacy may be needed to determine if Amiselimod may elicit therapeutic effects. Together, these studies strongly suggest therapeutic implications for S1PR modulation in IBD, perhaps with improved efficacy against UC as compared to CD.

## 5. Conclusions and Final Remarks

Altered sphingolipid metabolism and aberrant expression of sphingolipid enzymes are highly implicated in IBD and CRC, and are being developed as key therapeutic targets. Translation from the bench to the bedside may pose unique challenges for sphingolipids and their metabolizing enzymes. Many sphingolipid mimetics have issues with solubility and delivery. Additionally, PK studies that have been conducted on sphingolipid inhibitors have revealed reduced stability in vivo and short plasma half-life [61,75,116]. Nanoliposomes are currently being explored to improve delivery and stability for several sphingolipid mimetics and inhibitors. Additional concerns for safety and efficacy may arise from the ubiquitous expression of sphingolipid enzymes. Although in many of the preclinical models sphingolipid inhibitors have not induced toxicity, the clinical trials that have been conducted have demonstrated the potential for dose limiting toxicity in patients. The last 20 years has seen a flourish of activity characterizing and defining the roles for sphingolipid enzymes and sphingolipids in cellular signaling and disease. Based on current clinical trials and the efficacy of preclinical models, the next 20 years will likely see a continued push for sphingolipids and their generating enzymes as therapeutic targets.

Multiple interventions have been generated to investigate the therapeutic potential for sphingolipids, as well as sphingolipid enzymes and receptors. Currently, SK1 and S1PR interventions demonstrate the highest efficacy in cell culture and animal models, as well as patient trials. Furthermore, there is a large gap in the literature for various interventions for animal models of IBD and spontaneous models of CRC. Despite the promise that many interventions pose, at present only one sphingolipid intervention, the S1PR modulator Ozanimod, has been FDA approved for patients with UC. The assessment of interventions in animal models, which may be able to push forward more elaborate tiers of investigation, may result in the expansion of novel treatments available to patients.

## Figures and Tables

**Table 1:** Application of sphingolipid metabolism interventions.

			Animal Model				
Intervention	Cell Culture	Patient Culture	IBD	CAC	CRC	CRC Graft	Patient Trials
GCS Inhibitors							
Genz	[42]						
Miglustat *	[42]			[42]			
Eliglustat *	[43]					[43]	
aCDase Inhibitors							
B13	[44]						
LCL-204	[45,46]						
LCL-521	[47,48,49]						
C-2	[50,51,52,53,54,55,56,57,58]						
nCDase Inhibitors							
C_6_ urea-Cer	[23,59]					[23]	
ACER Inhibitors							
D-e-MAPP	[60]						
SK1 Inhibitors							
LCL-351	[61]		[61]				
PF-543	[62,63]		[64]		[63]		
RB-005	[65]						
SK2 Inhibitors							
ABC294640	[66,67]		[68,69]	[70]			[71]
Cer Analogues							
C_2_-Cer	[72,73,74]						
LCL-29	[75,76,77,78]						
LCL-30	[79]					[80]	
S1PR Modulators							
FTY-720	[81,82]	[83]	[84,85,86,87,88,89,90,91]			[92]	
Ozanimod ^#^							[93,94]
Amiselimod							[95]

A summary of sphingolipid analogues, inhibitors, and modulators used and their respective models. References listed in order of appearance. Cell culture studies include non-CRC cell lines. * These therapies are FDA-approved medications for the intended use in type 1 Gaucher disease, and are currently investigated for efficacy in other neurological diseases. ^#^ This modulator is an FDA-approved medication for the intended use in multiple sclerosis and UC.
